# Research progress on incomplete partition type 3 inner ear malformation

**DOI:** 10.1007/s00405-024-08555-7

**Published:** 2024-03-18

**Authors:** Kaifan Xu, Yun Xiao, Jianfen Luo, Xiuhua Chao, Ruijie Wang, Zhaoming Fan, Haibo Wang, Lei Xu

**Affiliations:** 1 Department of Otolaryngology-Head and Neck Surgery, Shandong Provincial ENT Hospital, Shandong University, Jinan, China; 2 Department of Auditory Implantology, Second People’s Hospital of Shandong Province, jinan, China; 3 Shandong Institute of Otorhinolaryngology, jinan, China

**Keywords:** Inner ear malformation, Cochlear implantation, Incomplete partition type III, POU3F4 gene

## Abstract

**Purpose:**

This review aims to provides a comprehensive overview of the latest research progress on IP-III inner ear malformation, focusing on its geneticbasis, imaging features, cochlear implantation, and outcome.

**Methods:**

Review the literature on clinical and genetic mechanisms associated with IP-III.

**Results:**

Mutations in the POU3F4 gene emerge as the principal pathogenic contributors to IP-III anomalies, primarily manifesting through inner ear potential irregularities leading to deafness. While cochlear implantation stands as the primary intervention for restoring hearing, the unique nature of the inner ear anomaly escalates the complexity of surgical procedures and postoperative results. Hence, meticulous preoperative assessment to ascertain surgical feasibility and postoperative verification of electrode placement are imperative. Additionally, gene therapy holds promise as a prospective treatment modality.

**Conclusions:**

IP-III denotes X-linked recessive hereditary deafness, with cochlear implantation currently serving as the predominant therapeutic approach. Clinicians are tasked with preoperative assement and individualized postoperative rehabilitation.

## Introduction

**Fig. 1:**
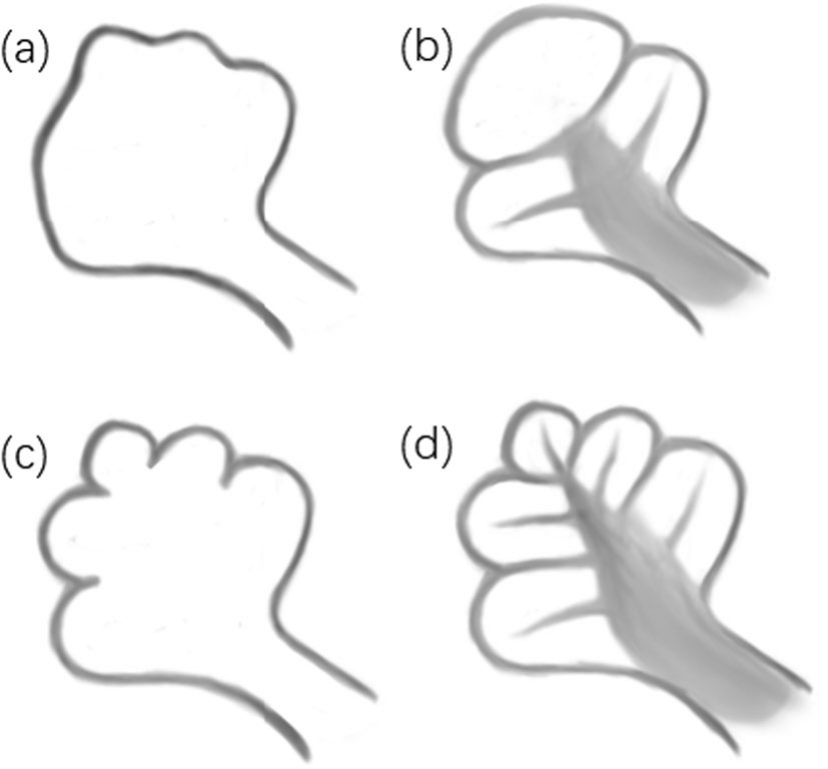
Incomplete cochlear partition deformity**a**,**b**,**c**with the normal cochlea**d**illustrated.**a**IP-I**b**IP-II**c**IP-III**d**normal cochlea

Inner ear malformations (IEM) were initially established in 1987 [1]. Phelps et al. provided the initial description of the HRCT findings related to this condition [2], and this distinctive deformity was first classified as an incomplete partition deformity in 2006 [3]. In a normal cochlea, there are interscalar septa between the inner wall of the cochlea and the modiolus, which divide the normal cochlea into 2½ or 2¾ turns encompassing the basal, middle, and apical regions [4]. Incomplete partition anomalies represent a group of cochlear malformations (Fig.1), where there is a clear differentiation between cochlea and vestibule, with normal external dimensions and various internal architecture defects. Among them, IP-I and IP-II exhibit a complete or partial absence of the interscalar septum [5]. IP-III is characterized by interscalar septa but lacks a complete modiolus on temporal bone CT [6]. Based on a previous study, it was determined that the external dimensions of the cochlea (height and diameter) in IP-III were comparable to those of a normal cochlea [7]. Therefore, IP-III is classified as an incomplete separation malformation based on the classification of inner ear malformations [4].

IP-III prevalence among cases of total inner ear anomaly is relatively low, accounting for approximately 0.9–2% of all cases [6]. Although IP-III mainly occurs in males, female carriers may also have late-onset hearing loss [8] and milder imaging findings [9]. It is estimated that X-linked nonsyndromic deafness contributes to only 1–5% of genetic nonsyndromic deafness, with X-linked deafness type 2(DFNX2) accounting for approximately 50% of all families with X-linked nonsyndromic deafness [10]. In this review, we summarize and discuss the pathogenic mechanisms, unique radiologic features, postoperative complications, and rehabilitation outcomes of IP-III caused by mutations in the POU3F4 gene. We will focus on the correlation between its genetic basis and clinical manifestations, aiming to provide new insights for further research on IP-III.

## Genetic basis of IP-III

### The structure of POU3F4

IP-III is primarily caused by mutations in the POU class 3 homeobox 4 (POU3F4) gene responsible for X-linked deafness type 2 (DFNX2). POU3F4 is a single copy gene located on the human chromosome X q21.1, spanning 3867 nucleotides, and the encoded transcription factor POU3F4 is a 361-amino-acid protein. The transcription factor POU3F4 binds DNA using a specific DNA-binding domain, which is divided into two subdomains, a POU-specific (POUs) and a POU homeodomain (POUHD) [11]. Most POU3F4 variants identified and reported in the literature are found within the sequence coding for one of the two DNA-binding domains. However, the correlation between the type of protein variant (truncation or amino acid substitution), the amino acid position of the truncation, or the type of amino acid substitution, and the severity of the disease (syndromic or non-syndromic) could not be found [12].

### The function of POU3F4

The POU3F4 gene is crucial for early embryonic development and is expressed in various tissues, including the otic capsule [13], brain [11], and pancreatic cells [14].Mutations in POU3F4 can cause severe mixed or sensorineural hearing loss, impacting all audiometric frequencies and potentially progressing over time [9]. To date, deafness is the only clinical manifestation. However, there have been reports indicating that children with mutations in this gene may have specific cognitive challenges, mental ill-health issues, and posture disorders [10, 15]. However, due to limited information, the role of chance factors must be considered.

The role of POU3F4 in the inner ear is indispensable. It collaborates with TBX1 in the growth and development of the cochlea, and the absence of this gene disrupts the gap junction plaques around the supporting cells [16], affecting the vascular pattern and the function of ion channels on the spiral ligament [17], thereby impacting the endolymphatic potential(EP)in the inner ear. Moreover, the silencing of this gene leads to downregulation of SLC6A20 in fibrocytes [18]. These studies reveal the basic principles of IP-III hearing loss. Besides, POU3F4-expressing otic mesenchyme cells promote spiral ganglion neuron survival in the postnatal mouse cochlea [19]. Together with EphA4, POU3F4 regulates spiral ganglion axon fasciculation, which is essential for appropriate auditory innervation [20]. Research has also shown that the overexpression of POU3F4 in non-neurogenic cortical astroglia can induce their transformation into neurons with electrophysiological activity [21]; POU3F4 has been found to promote the neuronal differentiation of neural stem cells (NSCs) via inhibition of CtBP2 [22]. POU3F4 regulates various aspects of inner ear growth and development and provides a potential tool for regenerating neurons in cell replacement therapy.

### Imaging features of IP-III

Computed Tomography (CT) and Magnetic Resonance Imaging (MRI) are essential for diagnosing IP-III inner ear malformation. its specific characteristics[23–25] are as follows:

### Middle ear

- The thickening of the stapes footplate is a prominent feature
- Round window atresia or underdevelopment may occur

### Cochlea and internal auditory canal (IAC)

- Bulbous dilation of the lateral end of the IAC
- Absence of the bony modiolus with interscalar septa present
- Hypoplasia at the cochlear base
- Thinning of the otic capsule
- irregular inner ear structure contours and hypodense areas in the otic capsule
- Enlargement of the vestibular aqueduct (EVA) has specific anatomic features and is seen in approximately 50% of cases.

### Vestibule and semicircular canals (SCC)

- Irregularly shaped vestibule with small cystic bulges in the margin
- Multicystic appearance of the SCC in some cases, with a widened lumen of the SCC in others
- Enlargement of the vestibular aqueduct (EVA) at the part close to the vestibule, with a cystic appearance present in some cases

### Nerve canals

- Markedly widened cochlear nerve canal
- Enlarged superior vestibular nerve canal
- Enlarged first part of the facial nerve in some cases

Additionally, MRI has revealed morphologic abnormalities in the hypothalamus [26] to some extent, indicating potential involvement of the central nervous system, which is consistent with previous reports of cognitive impairments observed in some patients.

The deformity of the cochlea is closely related to the function of the auditory nerve and postoperative outcomes. However, due to the absence of internal bony structures in IP-III deformities, MRI only shows inconsistent signals within the cochlea [27]. Additionally, the cochlea of animal models may not be entirely identical to that of humans. The pathologic reports on IP-III cochlear deformities in humans are still lacking.

### Cochlear implantation in IP-III patients

Cochlear implantation has emerged as a viable treatment option for individuals with IP-III inner ear malformation, offering the potential for improved hearing and speech recognition. However, surgical challenges and variable outcomes have been reported in this population.

### Surgical challenges

Cochlear implantation in patients with IP-III can be challenging due to the abnormal anatomy of the inner ear. Two central problems can occur during surgery: CSF gusher as round window approach and electrode misplacement into the IAC. Due to the absence of a bony partition between the basal turn of the cochlea and the IAC, the CSF will flow out into the middle ear cavity during CI surgery[28–30],which could potentially lead to continuous CSF leakage, CSF rhinorrhoea, meningitis, and intracranial infections, even cause electrode displacement, increasing the risk of secondary surgery. There is a risk of aberrant electrode array insertion into the internal auditory canal because of the absent modiolus and enlarged fundus of the IAC [28].

Some authors recommend creating a large cochleostomy to provide better access for packing and sealing the expected gusher [28, 31]. Enlargement of the cochleostomy also allows for visualization of the basal turn, the outer anti-modiolar wall, and the fundus of the IAC, facilitating the electrode array placement [29]. Position monitoring with intraoperative imaging can assist in*promptly*adjusting the angle and position of the inserted electrode [32].

The probability of longer electrodes entering the IAC is bigger; therefore, shorter electrodes are recommended to achieve full insertion and full stimulation [33]. The full-banded electrodes would be very risky because of facial nerve stimulation. So, half-banded electrodes are preferred [29]. Implant-related meningitis has received corresponding attention. Covering the patient with vaccination and firm sealing of the cochleostomy to stop the CSF leak can prevent the risk [34].

Tekin et al. has reported a case involving an IP-III cochlear patient who underwent robotically assisted cochlear implantation surgery (RACIS), and which resulted in a significant improvement of speech perception. They posit that RACIS uses data, sometimes beyond human and speech perception has improved perception, to warrant safety and accuracy [33]. RACIS has demonstrated the perfect application of artificial intelligence in highly sophisticated surgeries. However, it is worth noting that whether RACIS is used or not, it is essential to carefully evaluate the technical feasibility of cochlear implantation in IP-III patients and to take appropriate measures to mitigate the risks associated with surgery.

### Outcomes of cochlear implantation

Cochlear implantation outcomes in IP-III patients vary widely, ranging from poor [35] to results comparable to pediatric CI recipients without inner ear malformation [36]. Several studies have reported favorable hearing outcomes [37, 38] following cochlear implantation in IP-III patients, including improved sound field thresholds and speech recognition. Research studies have indicated that subjects with a truncation or deletion mutation achieved poorer outcomes than implants without Inner ear malformations (IEM) at 2 years post-CI [39].

Furthermore, the calculated electric load was significantly higher in IP III patients 1 year after CI. At 3 years following CI, pulse width and electric load were significantly greater in IP III patients. After violating the otic capsule, the assumed fibrosing process could induce higher impedances over time [31]. A study has shown that patients with IP-III demonstrate a decline in consonant recognition ability after three and a half years [40], which may be related to neuronal function. The absence of bony anatomic structures in the cochlear modiolus, as well as the gene's relevant effects on neurons, both contribute to increased susceptibility to spiral ganglion neurons (SGN) damage and reduce its repair capacity after injury. Besides, factors such as the degree of hearing loss, age at implantation [23], and postoperative rehabilitation [41]may influence the success of cochlear implantation in this population.

## Conclusion

Incomplete partition type 3 inner ear malformation is a rare genetic condition subtype of X-linked deafness. Advances in diagnostic imaging and genetic testing have greatly improved our understanding of IP-III and its underlying genetic basis. Cochlear implantation offers a promising treatment option for IP-III patients, although surgical challenges and variable outcomes need to be considered.

Efforts are also being made in the field of hearing aid therapy for congenital deafness.A study suggests that when bone thresholds are partially preserved, stimulation through bone or hybrid modalities, such as Varese bone–air stimulation, associated with proper rehabilitation, can help avoid subjecting young children to surgery burdened by potential serious complications while achieving similar communication outcomes [42].

Further research is also needed to better understand the cognitive functioning and mental ill-health issues in IP-III patients and to develop strategies for optimizing cochlear implantation outcomes in this unique population.

## Outlook

In a paper, based on the unique deafness mechanism of this gene, gene therapy may not be able to reverse the formed cochlear malformation. However, it may offer a certain degree of compensation regarding hearing [43]. If it works, this strategy could offer a new hope for improving the quality of life of DFNX2 children and their families.However, because of the blood–labyrinth barrier and the concealed anatomic location of the cochlea, the most common methods of inner ear drug delivery include administration through the round window membrane and cochleostomy. Non-invasive inner ear drug delivery is still in the early stages of basic research; it is a significant challenge in current clinical practice.

The exosomes have demonstrated extraordinary capabilities in drug delivery, with existing literature reporting successful penetration of the blood–brain barrier [44] and serving as gene carriers for Cas-9 gene therapy in the liver [45]. Exosomes also possess corresponding anti-inflammatory protective mechanisms [46] and may become a powerful tool in inner ear gene therapy.
